# Multi-ancestry genome-wide association study in *all of Us* for primary open-angle glaucoma

**DOI:** 10.1038/s41598-026-43993-9

**Published:** 2026-03-17

**Authors:** Kiana Tavakoli, Bonnie B. Huang, Tara Mirmira, Nichole Ma, Robert N. Weinreb, Sally L. Baxter

**Affiliations:** 1 Division of Ophthalmology Informatics and Data Science and Hamilton Glaucoma Center, Viterbi Family Department of Ophthalmology and Shiley Eye Institute, University of California San Diego, La Jolla, CA USA; 2 Division of Biomedical Informatics, Department of Medicine, University of California San Diego, La Jolla, CA USA; 3 Department of Computer Science and Engineering, University of California San Diego, La Jolla, CA USA; 4 Department of Medicine, University of California San Diego, La Jolla, CA USA; 5 9415 Campus Point Dr, 92093 La Jolla, CA USA

**Keywords:** Glaucoma, Open-Angle, Genetic Association Studies, Genetic Loci, Diseases, Genetics

## Abstract

This study aims to identify new genetic loci associated with primary open-angle glaucoma (POAG) and explore shared genetic risk factors across African, European, and Admixed American/Latino populations. Genome-wide Association Study (GWAS) utilizing data from the *All of Us* Research Program. The study included 374,254 participants, with 4,305 individuals diagnosed with POAG and 369,949 controls. Participants were categorized by ancestry: European, African, and Admixed American/Latino. We used short-read sequencing data and applied strict quality control measures (MAF > 0.01, INFO > 0.8). GWAS were conducted for each ancestry group using a logistic mixed model, adjusting for age, sex, and the top 11 principal components. A fixed-effect meta-analysis combined the results across ancestries. Genome-wide significance was set at *p* < 5 × 10^− 8^. The primary outcome measures were the identification of genetic loci associated with POAG, and the analysis of transcription factors linked to these loci in relevant tissues. In the European cohort, we identified four novel loci associated with POAG, linked to the *TUT4*,* RYK*,* MOXD1*,* and UBAP2* genes, as well as the previously known *TMCO1* locus. In the African cohort, we found five new loci, including *TSPAN17*,* SLC16A7*,* LOC100506869*,* LINC02388*,* and LOC107984606*. For the Admixed American/Latino cohort, we identified *GATA5*,* FAM135B*,* and LINC00871* genes as novel loci. Our analysis identified three novel loci in individuals of European ancestry, mapped to the genes *TUT4*,* RYK*,* and MOXD1*. We identified 56 genome-wide significant variants, including six putative novel loci, and found that most ancestry-specific signals replicated in the cross-ancestry meta-analysis, with the exception of several attenuated associations in the smaller Admixed American/Latino cohort. These findings indicate that the genetic determinants contributing to POAG may differ across populations, underscoring the importance of accounting for population-specific genetic architectures in the study of complex traits. Given the substantial variation in POAG prevalence among ancestries, it is plausible that certain genetic variants exert ancestry-specific effects. Consequently, conducting ancestry-stratified GWAS is essential for elucidating these unique genetic contributions.

**Supplementary Information:**

The online version contains supplementary material available at 10.1038/s41598-026-43993-9.

## Introduction

Primary Open Angle Glaucoma (POAG) is the leading cause of irreversible blindness globally^1^. It is a degenerative disease of the optic nerve that leads to progressive vision loss^2^. The transferability of genetic findings between populations is understood to be limited by ancestry-specific differences in linkage disequilibrium, minor allele frequency, and potentially differences in causal variants, which pose significant limitations to our understanding of the genetic architecture of POAG in non-European populations. This disparity may result in unequal benefits among different populations from precision medicine, as genetic risk models derived from large-scale studies conducted in European populations exhibit high predictive power in European samples but demonstrate poor predictive accuracy in non-European samples^3^. Consequently, enhancing ethnic and ancestral diversity among study participants is crucial for identifying understudied mechanisms of disease and ultimately ensuring equitable genetic findings^4,5^. Specifically, in large studies that focus only on people of European ancestry, disease-critical genetic variants may be missed because they are either rare or completely absent. In this study, we report a genome-wide association study (GWAS) of POAG utilizing the *All of Us*Research Program dataset, a diverse nationwide database in the United States that emphasizes the recruitment of populations historically underrepresented in biomedical research^6^. Our analysis includes individuals of European, African, and admixed American/Latino ancestries. We provide a comprehensive discussion on the identification of novel loci associated with POAG and examine the extent to which genetic signals are shared across ancestries, as well as the presence of ancestry-specific genetic signals. Our findings offer valuable insights into the etiology of POAG and underscore the importance of conducting genetic studies within non-European populations.

## Methods

### Study cohort

Data were sourced from the *All of Us*Research Program, a landmark research initiative aimed at advancing precision medicine by collecting and analyzing health data from diverse populations^6^. The program encompasses demographic, geographic, and medical diversity, including historically underrepresented populations such as ethnic minorities and individuals from underserved communities. All participants provided written informed consent, demonstrating their voluntary participation in the study and understanding of its purpose. Data sources for the *All of Us* Research Program include electronic health records, physical measurements, surveys, biospecimens, and wearable technology data.Prospective enrollment and data collection were approved by an independent institutional review board, with written informed consent obtained from all participants. The *All of Us* Data Research Center harmonized the data into the Observational Medical Outcomes Partnership (OMOP) common data model, a standardized framework for representing observational health data from diverse sources. To protect participant privacy, the Data Research Center applied measures such as deidentification and date shifting before making the data available on the *All of Us*Researcher Workbench. Secondary analyses of these deidentified datasets were classified as not involving human subjects research by the University of California San Diego Institutional Review Board. This study was conducted in accordance with the Declaration of Helsinki and followed the STROBE (Strengthening the Reporting of Observational Studies in Epidemiology) guidelines for observational research^7^..

### Phenotyping

There were 403,916 individuals who were enrolled in *All of Us* and had short-read sequencing data available on the *All of Us*Researcher Workbench Controlled Tier dataset version 8. Participants diagnosed with POAG were identified using SNOMED concept ID 77,075,001 (“Primary open angle glaucoma”) derived from electronic health record data. Individuals with normal-tension glaucoma, a subtype of glaucoma where optic nerve damage occurs despite normal intraocular pressure (IOP), were excluded from the study to ensure a homogeneous cohort. Formal, published validation rates (e.g., false positive/negative rates, PPV) for this specific SNOMED-based POAG algorithm within the AoU dataset are not publicly available. However, previous research in other large biobanks and healthcare systems indicates that phenotype algorithms requiring multiple data points (e.g., multiple codes, medication data) tend to have high positive predictive value (typically > 90%), but their sensitivity (recall) can vary^8–10^. Participants lacking age, sex, or genotype data were excluded as these individuals would not possess the expected covariates for the association analysis. Categories of genetically-determined ancestry in *All of Us*corresponded directly to categorical ancestry definitions used within gnomAD^11^, the Human Genome Diversity Project^12^, and 1000 Genomes^10^: African/African American, Admixed American/Latino, East Asian, European, Middle Eastern, South Asian, and Other (meaning an individual’s predominant ancestry is < 50% of their total ancestral composition).

### Genotyping

Details of genotyping procedures used by All of Us have been described previously11. We excluded variants with a call rate below 99% or with fewer than five heterozygous genotype calls. Therefore, the filtering criterion was applied at the variant level, not the sample level. No imputation was required in the All of Us research dataset, as data was generated from short-read whole genome sequencing across 403,916 individuals. Genomic analysis used the GRCh38 reference genome^15^. We utilized the Allele Count Allele Frequency (ACAF) data in the All of Us Researcher Workbench. The ACAF threshold callset includes variants with a population-specific allele frequency (AF) greater than 1% or a population-specific allele count over 100 in any ancestral subpopulations. Quality control measures ensured genotype data reliability, including filtering out variants with allele frequency < 1% and applying a stringent Hardy–Weinberg Equilibrium filter (*p* < 1 × 10⁻¹⁰), which is commonly used in large whole-genome sequencing studies to remove variants likely representing technical artifacts rather than true biological deviation. As recommended, we also repeated the analysis using an HWE threshold of *p* < 1 × 10⁻⁶, and the results were unchanged. (Figure S1)^16^. Logistic regression analysis was conducted separately for each autosome, adjusting for covariates including sex, age, and the top 11 genotyping principal components, which were selected based on scree-plot patterns and visual inspection of ancestry clustering in the PCA (Figure S2). The variance explained by each component showed a clear drop after PC11, beyond which additional PCs contributed minimally to population structure and did not capture meaningful ancestry-related variation. In parallel, the scatter plots for PCs 1–11 demonstrated clear separation of major ancestry groups (AFR, EUR, AMR), while higher PCs reflected noise rather than structured variation.To confirm that this choice did not influence results, we performed sensitivity checks using fewer (5) and more (15) PCs. The findings were unchanged, supporting the robustness of selecting 11 PCs Related individuals were excluded using pairwise kinship estimates derived from the srWGS data. Samples with kinship coefficients > 0.1 were considered closely related, and to ensure independence of observations, one individual from each related pair was randomly retained. The kinship coefficient reflects half of the proportion of shared genetic material (e.g., parent–child and full siblings have kinship ≈ 0.25; identical twins ≈ 0.5). Each pair is reported only once (e.g. sample1, sample2, 0.25} is equivalent to {sample2, sample1, 00.25) and self-kinship values are not reported. We avoided categorical classifications such as ‘first-degree relatives’ and instead relied directly on the kinship coefficients. While a mixed-model approach may be considered for retaining related individuals while controlling for relatedness, the analytic framework of our study required the removal of closely related samples to ensure independence of observations. Random exclusion of one individual per related pair was performed only among pairs exceeding the kinship threshold (> 0.1). We also quantified the impact of these exclusions on the cohort and applied alternative filtering approaches as sensitivity analyses, including retaining all individuals and modeling relatedness. (https://support.researchallofus.org/hc/en-us/articles/29475228181908-How-the-All-of-Us-Genomic-data-are-organized)

Our analysis utilized *Hail*^17^ for scalable genomic data analysis, *Bokeh*^18^ for interactive visualization, *Pandas*^19^ for data manipulation, and *NumPy*^20^ for numerical computing.

### Genome-wide association study

To account for potential population stratification amongst our study participants within ancestry categories, we projected everyone’s genotype by principal components using cohort-wide standardized genotypes.

We performed ancestry-specific GWAS analyses for each group of European, African, and Admixed American/Latino ancestries to explore genetic associations unique to each ancestry. For each population, we separately computed the top 11 genotyping PCs. To this end, we performed a logistic regression Wald test. Manhattan and quantile-quantile plots were generated to visualize the GWAS results and compute the genomic inflation factor, which could reveal unaccounted population stratification (lambda = 1.00). Genome-wide significant single nucleotide polymorphisms (SNPs) were identified at a threshold of *p* < 5 × 10^− 8^for each ancestry group and separately for the cross-ancestry meta-analysis^21^..

We defined a POAG-associated locus as a genomic region within ± 1 Mb of the lead variant. A locus was considered novel if it did not include any previously reported variants with a p-value < 5 × 10^− 8^in previous GWAS nor was in high linkage disequilibrium (r² >0.1) with genome-wide significant POAG variants from previous GWAS^21^. We employed the GWAS Catalog^22^ and Litvar^23^ databases to account for previous GWAS. If a genome-wide significant SNP landed in a protein-coding region of a gene, we also searched the GWAS Catalog to identify if this gene was associated with any potential comorbidities which may be physiologically connected with POAG. To evaluate the reproducibility of our ancestry-specific association signals, we investigated replication in an independent African ancestry cohort. Lead variants identified in the *All of Us* African ancestry analysis were shared with the Primary Open-Angle African American Glaucoma Genetics (POAAGG) study team, who provided association results for each locus using their internal GWAS pipeline. Although none of the tested variants achieved statistical significance in this external dataset, differences in phenotype definition, allele frequencies, and cohort size may have reduced replication power.

For the AMR ancestry, no suitable external cohort with comparable phenotype definitions and sequencing depth was available for replication. We note this as a limitation of the present study. To strengthen reliability despite the lack of external AMR validation, we performed internal sensitivity analyses using alternative covariate sets and QC thresholds, all of which yielded consistent association patterns. To assess whether the imbalance in age and sex between cases and controls could introduce residual confounding or collider bias, we performed an age and sex matched sensitivity analysis. For each POAG case, we randomly selected ten controls matched on sex and age. The complete GWAS workflow, including QC procedures, covariate adjustments, and association testing, was repeated using this matched dataset.

To address the need for independent validation, we systematically evaluated all lead variants in publicly available POAG GWAS datasets, including UK Biobank (European ancestry), POAAGG (African ancestry), and other published studies identified through the GWAS Catalog. For each lead SNP, we assessed exact replication, linkage disequilibrium–based concordance (r² ≥ 0.6), and direction of effect consistency.(Table S1).

### Fixed-effect meta-analysis and multi-ancestry GWAS

We conducted a fixed-effect meta-analysis across three ancestry groups (European, African, and Admixed American/Latino) by integrating summary statistics from separate GWAS for each group. The remaining ancestry groups available in *All of Us* were not included in these analyses due to prohibitively small sample sizes.

We applied an inverse-variance-weighted fixed-effect meta-analysis to these ancestry-specific results, which enhanced our overall statistical power to identify POAG-associated variants. We estimated meta-analyzed effect sizes and standard errors for each variant and calculated p-values based on a normal distribution. This method integrated data from multiple ancestry groups, providing a comprehensive view of genetic associations that may be shared across populations.

### Study cohort characteristics

We identified 4,305 cases of POAG and 369,949 controls without POAG. Among these participants, there were 2,302 cases of European ancestry, 1,339 cases of African ancestry, and 465 cases of Admixed American/Latino ancestry. (Table 1)

**Table 1:** Demographic and clinical characteristics of All of Us participants with and without primary open-angle glaucoma (POAG) who had genotype data available for analysis. This table summarizes the baseline characteristics of the analytic cohort, including sex, age, self-reported race and ethnicity as recorded in the electronic health record (EHR), and POAG case–control status. The Hispanic/Latino variable reflects self-reported ethnicity in the EHR and is presented solely for descriptive purposes. Association analyses relied exclusively on genetically inferred ancestry.

Mean (Standard Deviation) Age	POAG Cases (*N* = 4,305)	Controls (*N* = 369,949)
	73.9 (10.6) years	56.17 (17.0) years
No. (%) Hispanic	572 (13.3% of cases)	70,329 (19% of controls)
No. (%) Male	2,195 (51% of cases)	146,223 (39.5% of controls)
No. (%) Female	2,110 (49% of cases)	223,726 (60.5% of controls)
No. (%) African ancestry	1,339 (31.1% of cases)	69,491 (18.8% of controls)
No. (%) Admixed American/Latino ancestry	465 (10.9% of cases)	67,875 (18.3% of controls)
No. (%) European ancestry	2,302 (53.5% of cases)	213,774 (57.8% of controls)
No. (%) Other ancestry	199 (4.5% of cases)	18,809 (5.1% of controls)

## Results

### European POAG GWAS identifies newly associated variants near genes with known roles in eye development and function

**Fig. 1:**
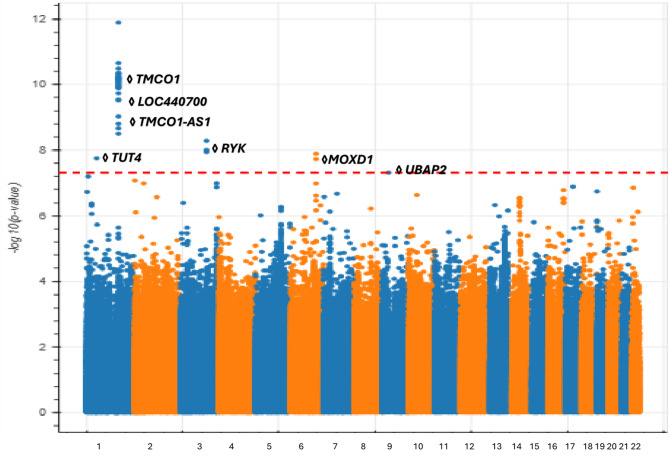
Manhattan plot of genome-wide association results for POAG in *All of Us* participants of European ancestry.The plot highlights all five genome-wide significant loci, including the four putative novel loci near TUT4 (Chr1), RYK (Chr3), MOXD1 (Chr6), and UBAP2 (Chr9), as well as the previously reported locus near TMCO1 (Chr1). Lead SNPs at each locus are labeled with the nearest gene. Genome-wide significance threshold (*P* = 5 × 10⁻⁸) is indicated by the horizontal dashed line.

The analysis of individuals of European ancestry (2,302 POAG cases, 213,774 controls) identified 52 genome-wide significant variants and five distinct loci associated with POAG, consistent with previous GWAS findings related to POAG and visual field loss^24^. Notably, four of these loci were novel and have not been previously reported for POAG or glaucoma in general.(Fig. 1)(Table S2).

On chromosome 1, we observed a significant number of associated variants near the *TMCO1*gene, which has been implicated in various disorders, including POAG, craniofacial dysmorphism, skeletal anomalies, and impaired intellectual development syndrome^25^.*TMCO1* plays a crucial role in regulating intraocular pressure (IOP), a key factor in the development of POAG. Dysregulation of *TMCO1*may hinder the outflow of aqueous humor potentially resulting in elevated IOP levels^26,27^. Additionally, on chromosome 1, we replicated the association near the pseudogene *LOC440700* and the *TMCO-AS1*gene which have both been previously reported in POAG GWAS^28,29^..

We identified several loci associated with POAG that have not been previously reported by existing GWAS. One such locus is centered at 52.5 Mb on chromosome 1 near the *TUT4*gene, which is responsible for uridylating miRNAs^30^. This gene is related to glutathione peroxidase 7, where changes in enzyme activity may contribute to age-related macular degeneration (AMD)^31,32^. Furthermore, *TUT4*has been linked to height^33^,with studies suggesting that individuals who are taller or have lower body mass index tend to have a smaller neuroretina rim area and a larger optic cup-to-disc area ratio^34^..

On chromosome 3, we discovered an associated locus consisting of intronic variants within the *RYK* gene. The *RYK*gene significantly influences eye development, particularly through its modulation of Wnt signaling pathways critical for eye organogenesis^35^. Additionally, *RYK*has been shown to affect systolic and diastolic blood pressure^36^,and numerous studies have demonstrated an association between blood pressure and POAG^37–39^..

We identified another POAG-associated locus centered on the promoter region of the *MOXD1* gene on chromosome 6 (at 132.2 Mb). *MOXD1* has been implicated in the progression of AMD^40^and anemia^41^.*MOXD1*is also known to affect tau protein levels, which may lead to modifications in neuronal injury associated with ocular hypertension^42,43^. Lastly we identified a POAG-associated intronic variant on chromosome 9 which encodes *UBAP2*, a gene associated with the neurodegenerative disease amyotrophic lateral sclerosis^44^,in which astrocytes play a role in both ALS disease and in changes to the optic nerve head in glaucoma^45^..

### African ancestry GWAS identifies new loci not previously identified with European GWAS data

**Fig. 2:**
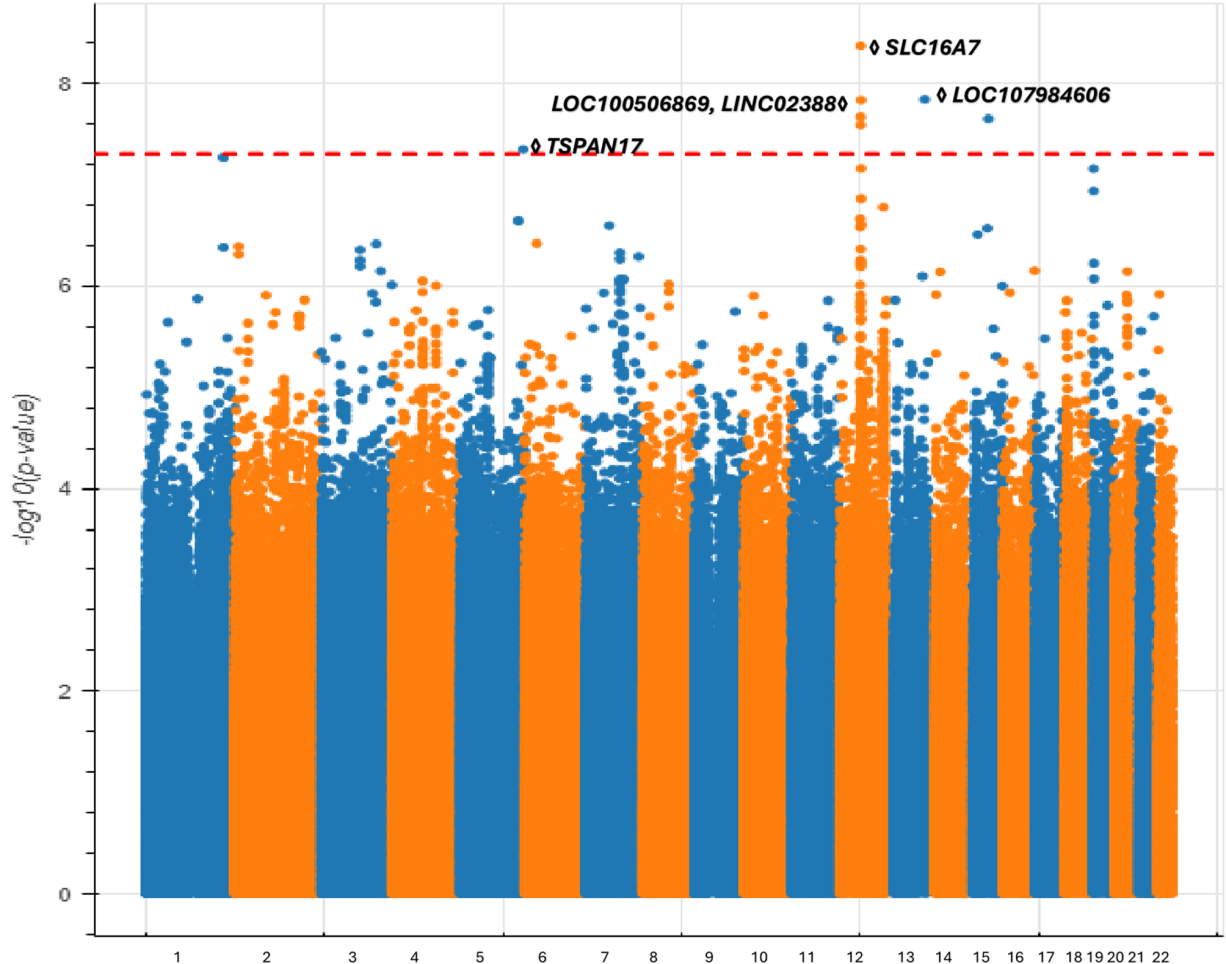
*Manhattan plot of genome-wide association results for POAG in* *All of Us* *participants of African ancestry.The plot highlights five independent genome-wide significant loci*,* all of which appear novel for POAG*,* including associations near TSPAN17 (Chr5)*,* SLC16A7 (Chr12)*,* LOC100506869/LINC02388 (Chr12)*,* LOC107984606 (Chr13)*, and a noncoding region on Chr15. Lead SNPs at each locus are labeled with the nearest gene. The genome-wide significance threshold (*P* = 5 × 10⁻⁸) is shown by the horizontal dashed line.

During our investigation into the genetic factors contributing to POAG within the African ancestry group (1,339 POAG cases, 69,491 controls), we uncovered novel associations that highlight the intricate genetic complexity and remarkable diversity present in POAG genes in different populations. Our research identified seven genome-wide significant SNPs across five independent loci (as determined by distance and linkage disequilibrium) (Fig. 2, Table S2).

None of these associations have been previously identified in any GWAS related to POAG. While this GWAS did not detect a genome-wide significant association at the well-established TMCO1 locus found in European POAG GWAS, prior studies have reported associations at TMCO1 in individuals of African ancestry. The absence of a significant signal in our analysis may reflect limited statistical power or differences in allele frequency across populations, rather than a definitive lack of involvement of TMCO1 in POAG pathogenesis in non-European individuals.

Here, we summarize these novel POAG-associated loci in order of genomic coordinates. First, we identified an associated locus on chromosome 5 centered on an intronic variant of the *TSPAN17* gene. The expression of *TSPAN17*in the neural tube and brain suggests a potential influence on neurological factors related to POAG^46^. Second, on chromosome 12, we identified another associated locus centered on the *SLC16A7*gene, which has been implicated in age-related cataract and is expressed in retinal tissue^47^. Notably, it has been shown that AMD and POAG exhibit a positive genetic correlation^48^. Additionally, previous work indicates that *SLC16A7* may affect alcohol consumption^49^, which has been shown to increase the risk of glaucoma^50^. Third, also on chromosome 12, but more than 1 Mb away, we identified a locus harboring two non-coding RNA genes: *LOC100506869* and *LINC02388*. The latter gene has been connected to cataract formation^51^,which may contribute to primary angle-closure glaucoma due to a narrower drainage angle in the eye. While cataracts do not directly cause glaucoma, there are rare instances where cataracts can lead to elevated IOP and damage to the optic nerve^52^..

Lastly, we discovered an associated locus on chromosome 13 encoding the *LOC107984606* gene with no immediate connection to POAG pathogenesis, as well as an association on chromosome 15 centered on a nonfunctional variant which does not encode any gene.

### *All of Us* cohort enables first POAG GWAS for individuals of Admixed American/Latino ancestry

**Fig. 3:**
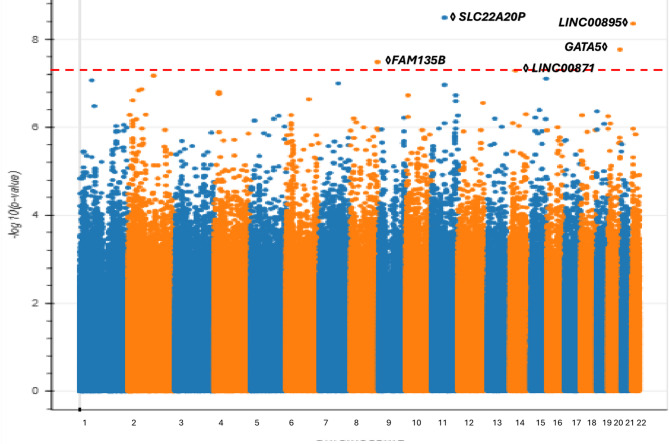
Manhattan plot of genome-wide association results for POAG in *All of Us* participants of Admixed American/Latino ancestry.The plot highlights five genome-wide significant loci, including a locus near SLC22A20P (Chr11) that is adjacent to, but only moderately correlated with, previously reported European POAG associations, and a locus near LINC00895 (Chr22).verma Additional putative novel loci include associations near FAM135B (Chr8), LINC00871 (Chr14), and GATA5 (Chr20). Lead SNPs at each locus are labeled with the nearest gene. The genome-wide significance threshold (*P* = 5 × 10⁻⁸) is indicated by the horizontal dashed line.

The modest sample size of the Admixed American/Latino population in our cohort (465 POAG cases, 67,875 controls) has enabled us to conduct an ancestry-specific GWAS for this demographic, whereas previous studies suffered from small sample size and thus were only powered to perform cross-ancestry meta-analysis^28^. Our advance toward learning population-specific genetic susceptibility for POAG is critical as Latinos are approximately 5% more likely to be affected by POAG compared to other populations^53^. Our analysis led to the identification of five genome-wide significant variants, constituting independent loci. (Fig. 3, Table S2).

First, we observed a locus on chromosome 11 in proximity to SLC22A20P, a region previously implicated in European POAG GWAS. Given the very low LD (r² < 0.1) with the reported variants, our signal likely represents a potentially independent ancestry-specific association rather than a replication^28,54^. The genome-wide significant variants from the European GWAS^25,51^ are in moderate linkage disequilibrium (r^2^ > 0.1) with our lead variant. The *SLC22A20P*gene has been shown to influence mean corpuscular hemoglobin levels^55^,with higher levels correlating with a faster rate of retinal nerve fiber layer (RNFL) thinning^56^..

Second, we identified a genome-wide significant locus on chromosome 22, near encoding intergenic the non-coding RNA gene *LINC00895*. This locus is situated within ± 1 Mb of previously associated POAG variants found in previous GWAS^54^, although these variants were suggested to regulate different genes. Importantly, the variants identified in these previous studies exhibited low linkage disequilibrium (r² < 0.1) with our lead variant, potentially suggesting that this finding may represent an independent mechanism. The *LINC00895*gene is known to affect platelet count, and lower platelet counts have been observed in individuals with POAG^57^..

Although these loci have not been previously reported as POAG-associated, they lie near genomic regions highlighted in earlier GWAS. Therefore, we refer to them as *putative novel loci* that occur adjacent to previously identified association signals and require replication to determine whether they represent independent effects or extensions of known loci.

We also identified several loci that have not previously been implicated in POAG. First, on chromosome 8, we identified an associated locus centered on an intronic variant of *FAM135B*which is associated with smoking behavior^46^. Second, we identified a genome-wide significant intronic variant of *LINC00871*on chromosome 14. Expression of this gene is observed in the basal ganglia, particularly within the caudate and putamen nuclei. Prior GWAS has reported the association of this variant with Sjögren’s syndrome, which has implications for ocular dryness^58^. Moreover, *LINC00871*has been associated with body mass index^59^, suggesting potential pleiotropy affecting obesity and the development of POAG^60^, as well as smoking status and initiation^61^highlighting the impact of tobacco use on POAG^62^..

Third, on chromosome 20, we identified a POAG-associated variant 35 kb upstream of the *GATA5* gene, which is associated with AMD. Others have hypothesized that the mechanisms underlying the associations at the *GATA5*locus in neovascular AMD patients may be linked to retinoic acid signaling^63^. Furthermore, *GATA5*has been shown to affect hematocrit levels^64^,potentially contributing to increased IOP^65^. Additionally, *GATA5*influences lung function^66^,where reduced lung function has been associated with an increased risk of glaucoma^67^..

### External validation of lead POAG loci

To evaluate external support for the loci identified in our analysis of individuals with American Latino ancestry, we assessed the lead variants in publicly available POAG GWAS datasets from other populations. Because large publicly available datasets in American Latino ancestry are currently limited, replication was performed using the UK Biobank European ancestry GWAS as well as previously published studies identified through the GWAS Catalog^51,53,54^. For each lead SNP, we evaluated the presence of the same variant in the external datasets and assessed statistical significance and direction of effect. When the exact variant was not available, nearby variants within the same locus (± 1 Mb) were examined to determine locus-level concordance.

The locus on chromosome 1 near *TMCO1* demonstrated strong external support in the UK Biobank European ancestry dataset, where multiple variants within this region showed highly significant associations with POAG and consistent direction of effect. This locus has been previously reported in several GWAS of POAG, further supporting its role in disease susceptibility.

In addition, loci on chromosomes 20 and 22, which were identified in our American Latino ancestry analysis, demonstrated significant associations within the same genomic regions in the European ancestry dataset. Although the exact lead variants were not always present in the external datasets, nearby variants within these loci showed statistically significant associations and concordant direction of effect, supporting locus-level consistency across populations.

For several additional loci, variants were either not present in the available summary statistics or did not reach statistical significance in the external datasets, although some demonstrated directionally consistent effects. These differences may reflect variation in linkage disequilibrium structure across populations, limited representation of American Latino ancestry in publicly available GWAS datasets, or differences in statistical power between studies.

A complete summary of the external validation analysis, including exact variant matches, nearby supporting variants, and direction of effect comparisons, is provided in Supplementary Table S1.

### Cross-ancestry GWAS meta-analysis

In our analysis, we identified 56 genome-wide significant variants, 6 of which were not identified in ancestry-specific POAG GWAS. All but five of the genome-wide significant ancestry-specific GWAS variants were additionally found to be significant in the cross-ancestry meta-analysis. The exceptions mostly included variants identified in the Admixed American/Latino GWAS, which has a substantially smaller sample size and thus lower contribution to the cross-ancestry meta-analysis.

**Fig. 4:**
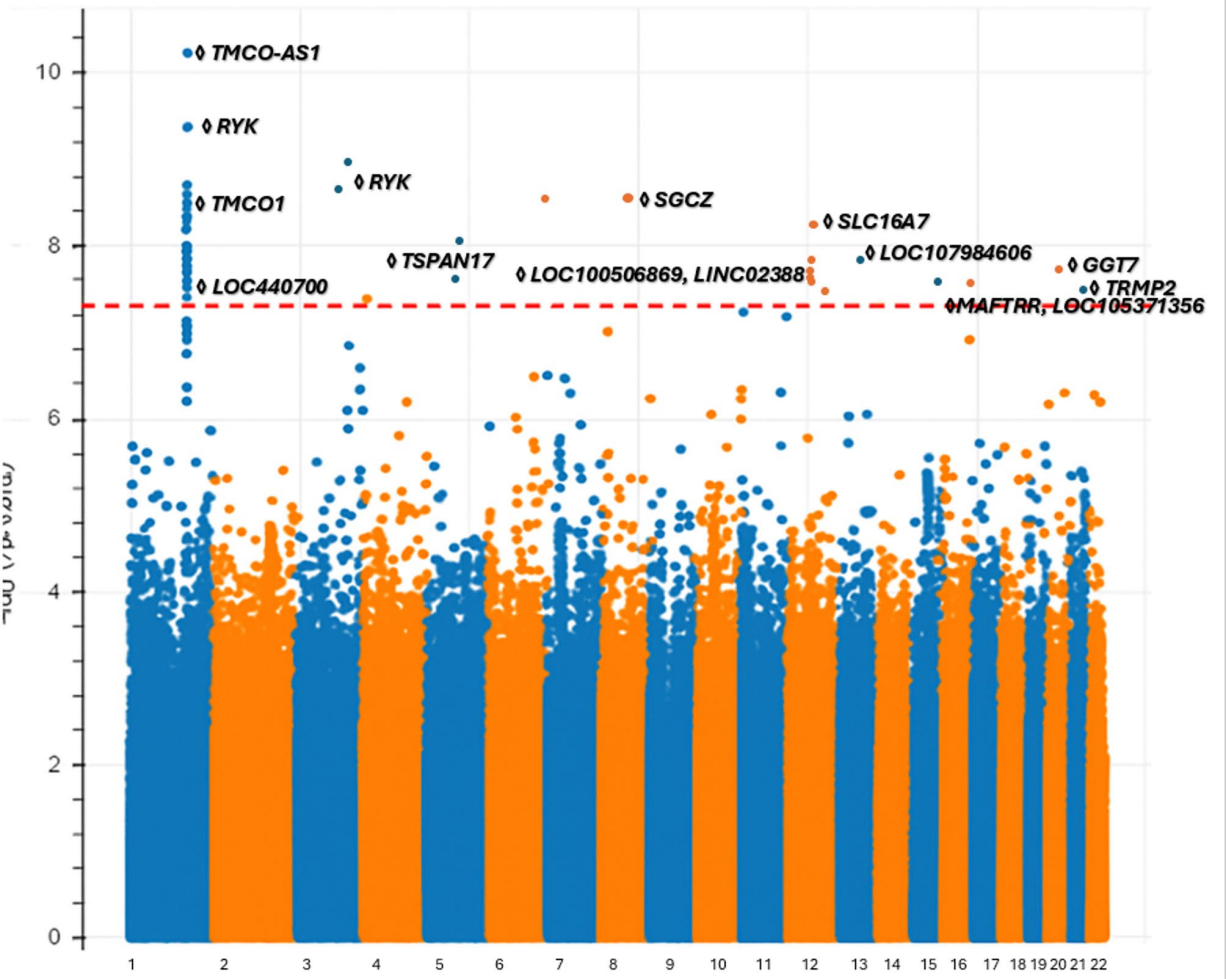
Manhattan plot of the meta-analysis GWAS for POAG.This Manhattan plot displays the genome-wide association results from the meta-analysis across all included cohorts. Each point represents a SNP plotted according to its chromosomal position (x-axis) and the –log₁₀(p-value) of its association with POAG (y-axis). Alternating colors denote adjacent chromosomes for visual clarity. The red dashed horizontal line indicates the genome-wide significance threshold (*p* = 5 × 10⁻⁸). Loci with SNPs surpassing this threshold are labeled with the nearest annotated gene or the most biologically plausible candidate gene based on regional context. All significant loci are highlighted in the figure and described in detail in the manuscript.

First, on chromosome 5, an intergenic variant was newly associated in the meta-analysis; the closest gene is ENSG00000286625 and is 10,000 Kb away. Second, we identified an intronic variant in the *SGCZ* gene on chromosome 8 that has previously been linked to BMI^68^, reinforcing the possible role of metabolic pathways in glaucoma development. Third, we detected an intronic variant on chromosome 12,the *SLC16A7* gene influencing body weight and BMI^49^, suggesting a relationship between metabolic factors and POAG risk^60^. Fourth, we identified one intronic variant on chromosome 16 in the *MAFTRR* and *LOC105371356*genes, both of which affect thyroid function, indicating a potential link between thyroid-related pathways and POAG susceptibility^69^. Fifth, on chromosome 20, we identified an intronic variant in the *GGT7*gene, which is linked to chronic kidney disease (CKD), suggesting a potential association between glaucoma and CKD^67^. Lastly, we identified a intron variant on chromosome 21,The gene *TRPM2*, which is a channel gene is associated with POAG, suggesting that TRPM2 may serve as a potential aqueous humor biomarker for glaucoma^71,72^.(Fig. 4 and Table S3).

Given the known variation in allele frequencies and LD patterns across global populations, we evaluated heterogeneity across ancestries for all genome-wide significant loci. We calculated Cochran’s Q and I² statistics, which are provided in Supplementary Table 3. Overall, the level of heterogeneity was modest for most loci. To further evaluate robustness, we additionally performed a random-effects meta-analysis, which yielded results highly consistent with the fixed-effect inverse-variance–weighted approach. No additional loci reached genome-wide significance under the random-effects framework, reinforcing the stability of the findings.

An important limitation of the present study is the substantial imbalance in case numbers across ancestry groups: European ancestry participants (2,302 cases) comprise the largest group, followed by African ancestry (1,339 cases), with considerably fewer cases among Admixed American/Latino participants (465 cases). As a result, power to detect associations varies substantially across ancestries. Loci identified in European ancestry participants may fail to replicate in African or Latino participants due to reduced statistical power, rather than true absence of effect. Conversely, ancestry-specific associations in African and Latino groups should be interpreted cautiously, as limited sample sizes increase the possibility of unstable effect estimates or false positives.

We explored trans-ethnic and multi-ancestry meta-analysis strategies; however, several ancestry-specific signals were markedly attenuated when combining populations, likely reflecting heterogeneity in allele frequencies and LD patterns. Therefore, we present both fixed-effect and random-effect meta-analyses, which offer a more stable cross-ancestry framework without substantial loss of signal strength. Although Bayesian fine-mapping can provide valuable insights, the modest sample sizes and limited number of genome-wide significant variants in some ancestry groups prevented generation of informative credible sets, and thus fine-mapping was not feasible in the current study.

The association results from the matched-analysis were highly consistent with our primary findings, showing no meaningful changes in effect sizes or significance patterns.

## Discussion

Our study highlights the benefits of conducting genetic research in non-European populations. LD often poses a significant challenge in identifying causal variants in GWAS. However, an analysis of GWAS results from different ancestries with diverse LD structures can enhance the precision of causal variant identification. We performed this analysis for African ancestry, as well as for European and admixed American Latino populations. While previous GWAS have included non-European populations, such as those studied in the DIGS/ADAGES and NEIGHBORHOOD consortia^3^, a large proportion of prior research has focused on European ancestry groups^73^. Based on our literature search, only one prior study has investigated POAG in admixed Latino populations^74^, highlighting the importance of exploring genetic contributions in these underrepresented groups. However, there remains a significant gap in our understanding of genetic risk factors in other admixed populations, despite the increasing incidence of POAG^75,76^ in these diverse communities. Additionally, our study identified novel loci and variants that have not been reported in earlier GWAS. The association results from the age- and sex-matched-analysis were highly consistent with our primary findings. This indicates that the observed associations are not driven by demographic imbalance and that residual confounding or collider bias related to age and sex is unlikely to explain the results. Both our sensitivity analyses and the application of alternative filtering thresholds yielded consistent results, supporting the stability of our findings.

Our analysis revealed three new loci in European populations associated with genes *TUT4*,* RYK*, and *MOXD1*. Additionally, we identified five new loci from the African ancestry GWAS, as well as four novel loci in Admixed American/Latino ancestry. These results suggest that the genetic effects contributing to POAG may vary between populations, highlighting the importance of considering population-specific genetic architectures in complex traits. Given the significant differences in POAG prevalence across ancestries, it is likely that certain variants have ancestry-specific effects. Therefore, it is crucial to conduct ancestry-specific GWAS to uncover these unique genetic contributions.

While we provide functional context for several loci identified in this study, including *TUT4*, *RYK*, *MOXD1*, *UBAP2*, *GATA5*, and others, we acknowledge that many of these interpretations rely on indirect evidence from related biological pathways or associations with non-ocular traits such as age-related macular degeneration, cardiovascular traits, or metabolic phenotypes. For several of these loci, no direct experimental evidence currently links them to POAG or ocular tissue biology. Accordingly, we have distinguished in the discussion between loci with known roles in ocular function or glaucoma-relevant pathways and those whose potential involvement is based on pleiotropic effects or shared molecular mechanisms. The functional connections for these novel loci should therefore be considered exploratory and hypothesis-generating. Future studies incorporating ocular tissue expression datasets, functional genomics, and experimental validation will be essential to clarify the potential role of these genes in POAG pathophysiology.

These results are consistent with the possibility that genetic effects contributing to POAG may differ across populations; however, these observations could also reflect limited statistical power or differences in allele frequency rather than definitive biological heterogeneity.

In our study’s limitations, we acknowledge the relatively modest sample sizes for African, East Asian, Admixed American/Latino, and Middle Eastern populations, which may hinder the robustness of our GWAS findings in diverse ancestries. Additionally, the lack of data in *All of Us*on visual field measurements and IOP restricts our ability to assess the effects of novel variants or loci on these established factors that are known to be associated with POAG. In addition, phenotyping using EHR diagnostic codes has known limitations^73^, but additional clinical data that may assist with more precise phenotyping, such as imaging, testing, and free-text notes, are currently not available in *All of Us*. Another limitation of our study is the filtering threshold applied to the genomic data (minor allele frequency < 1% or allele count < 100). This decision was necessary to ensure adequate statistical power for the common-variant association tests performed here but resulted in a potential bias toward detecting common variants, primarily those prevalent in majority populations. Consequently, our findings may underrepresent the impact of rare variants, which are known to play a critical role in complex traits within diverse populations. We did not perform dedicated, ancestry-stratified rare variant analyses (such as burden or Sequence Kernel Association Test) in this work. We highlight this as an important area for future investigation.

In response to the need for independent validation, we systematically evaluated all lead loci across available POAG GWAS papers and datasets. The TMCO1 locus demonstrated robust external support in UK Biobank, with multiple lead variants showing exact replication and highly significant associations. Additionally, loci on chromosomes 20 (*GATA5* region) and 22 (*LINC0089*5 region), Native American/Latino ancestry checked on the European dataset, exhibited significant locus-level concordance with consistent direction of effect, further supporting their relevance in POAG susceptibility.

Several additional loci showed directionally consistent effects but did not reach statistical significance in external datasets. Differences in replication across loci may reflect ancestry-specific LD structure, variation in imputation coverage, sample size differences, and heterogeneity in phenotype definition across cohorts. Notably, lack of replication in publicly available datasets does not necessarily exclude biological relevance, particularly for variants that may be ancestry-specific or underpowered in prior studies.

A subset of loci could not be evaluated in external datasets due to absence of corresponding variants in available summary statistics. These findings may represent potentially novel POAG-associated regions that warrant further investigation in larger multi-ancestry cohorts.

We acknowledge that potential systematic biases in clinical diagnosis, documentation behavior, and access to care could lead to differences in case ascertainment rates across participants of diverse ancestries. While our study design and statistical methods for association testing account for population structure to mitigate confounding, these inherent EHR biases may impact the overall power and generalizability of the findings across different ancestral groups. This is a known limitation of using real-world EHR data, and our results should be interpreted with this context in mind.

Our study marks a significant advancement in understanding the genetic aspects of POAG across diverse populations. The findings provide insights into the genetic architecture of POAG, emphasizing the importance of genetic diversity in understanding disease susceptibility. Addressing challenges through more inclusive research that includes clinical, environmental, and genetic data is essential for developing effective, personalized interventions. Ongoing research is needed to validate these findings and clarify the functional consequences of identified genetic variations, ultimately aiming to improve early detection and management of this sight-threatening condition. Future studies will focus on the functional validation of these novel loci, including the integration of ocular-specific eQTL and gene expression data to elucidate the precise mechanisms, contribute to glaucoma pathogenesis. Another limitation of this study was that we were unable to generate locus-specific association plots that would have been valuable for visualizing LD structure and gene context around lead variants. However, generating these plots would require access to ancestry-matched LD reference panels, which are currently not available in the *All of Us* Researcher Workbench. This may be available in future versions of the dataset and represents another area for future investigation.

A major limitation of this study is the lack of replication in independent cohorts. Independent replication is critical for validating genome-wide significant associations and reducing the risk of false-positive findings. Despite efforts to identify suitable external datasets, the availability of publicly accessible datasets with comparable phenotypes, ancestry representation, and complete summary statistics was limited. This restricted our ability to perform independent replication analyses within the scope of the current study. Accordingly, the reported associations should be interpreted with caution and considered hypothesis-generating, pending validation in future studies leveraging additional publicly available or consortium-based datasets.

These ancestry-specific associations have important implications for genetic risk prediction and health equity. Because most existing POAG polygenic risk scores have been derived largely from European-ancestry cohorts, their transferability to other populations remains limited. Our findings highlight that certain loci appear shared across ancestries, whereas others are distinct or attenuated in multi-ancestry analyses, suggesting that population-specific genetic architecture contributes meaningfully to POAG susceptibility. Such heterogeneity may complicate cross-ancestry PRS performance and underscores the need for ancestry-diverse training datasets to improve calibration and reduce disparities in genetic risk prediction. Furthermore, several of the loci identified here overlap or fall near signals reported in prior global glaucoma GWAS, while others may reflect novel ancestry-specific contributions, reinforcing the value of expanding genomic studies in underrepresented populations.

## Supplementary Information

Below is the link to the electronic supplementary material.

Supplementary Material 1

Supplementary Material 2

Supplementary Material 3

Supplementary Material 4

Supplementary Material 5

## Data Availability

This study was conducted using data from the *All of Us* Research Program. The data are available to registered and contolled researchers through the *All of Us* Researcher Workbench. Access to individual-level data is controlled and requires approval by the *All of Us* Research Program.

## Abbreviations

POAG: Primary open-angle glaucoma
GWAS: Genome-Wide Association Studies
IOP: Intraocular Pressure
AMD: Age-related Macular Degeneration

## Declarations

### Meeting presentation

Paper presentation at the 2024 American Academy of Ophthalmology (AAO) Annual Meeting and poster presentation at 2024 American Society of Human Genetics (ASHG) Annual Meeting, Presentation at 2025 World Glaucoma Congress.
