# Supplementary material for: Multi-ancestry genome-wide association study in all of Us for primary open-angle glaucoma
Source: Sci Rep. 2026 Mar 17;16:13788. doi: 10.1038/s41598-026-43993-9 (PMC13129092; doi:10.1038/s41598-026-43993-9)
Supplement: Supplementary file 1 — Supplementary Material 1 [file 41598_2026_43993_MOESM1_ESM.pdf]

Quality Control workflow diagram

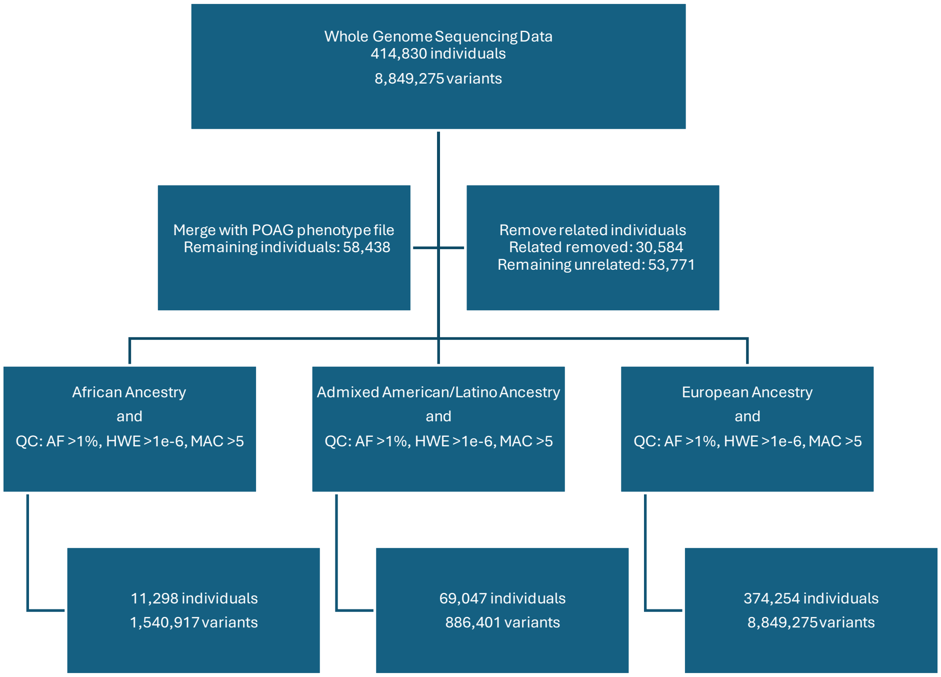

Quality control workflow for All of Us whole-genome sequencing data. After phenotype merging and relatedness removal, ancestry assignment was performed using AOU PCA-based predictions. Variant-level QC included HWE filtering, MAC thresholds, and AF filters. The diagram shows the final number of individuals and variants for African (AFR) ,admixed American/Latino (AMR) ancestry, and European (EUR) ancestry groups. This is after the sensitivity analysis.
