# Supplementary material for: Multi-ancestry genome-wide association study in all of Us for primary open-angle glaucoma
Source: Sci Rep. 2026 Mar 17;16:13788. doi: 10.1038/s41598-026-43993-9 (PMC13129092; doi:10.1038/s41598-026-43993-9)
Supplement: Supplementary file 2 — Supplementary Material 2 [file 41598_2026_43993_MOESM2_ESM.pdf]

Supplementary Figure 2

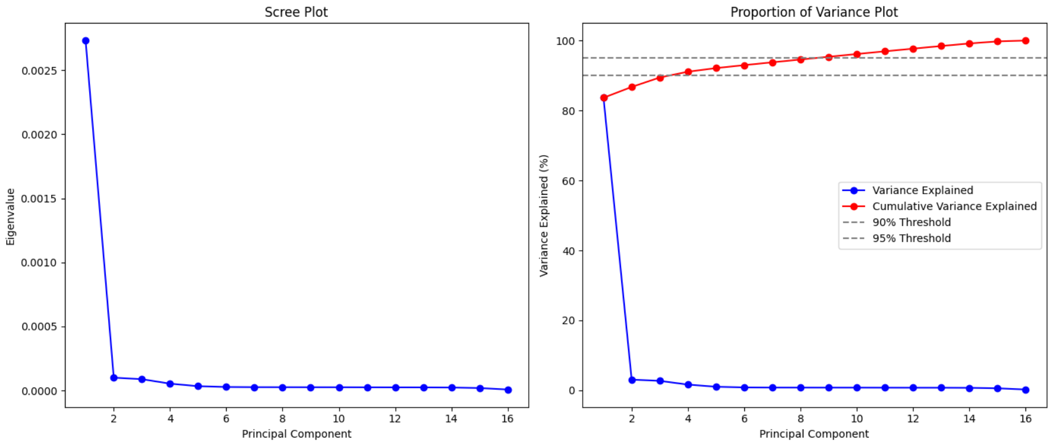

African Ancestry PC features plot

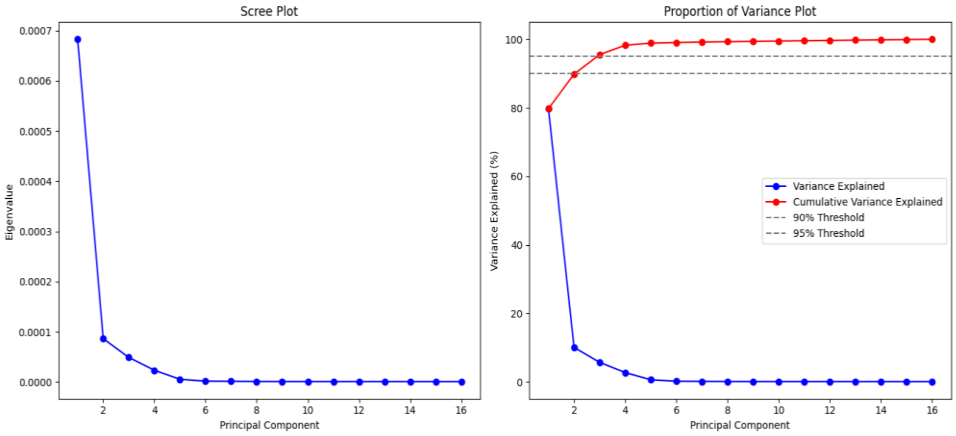

European Ancestry PC features plot

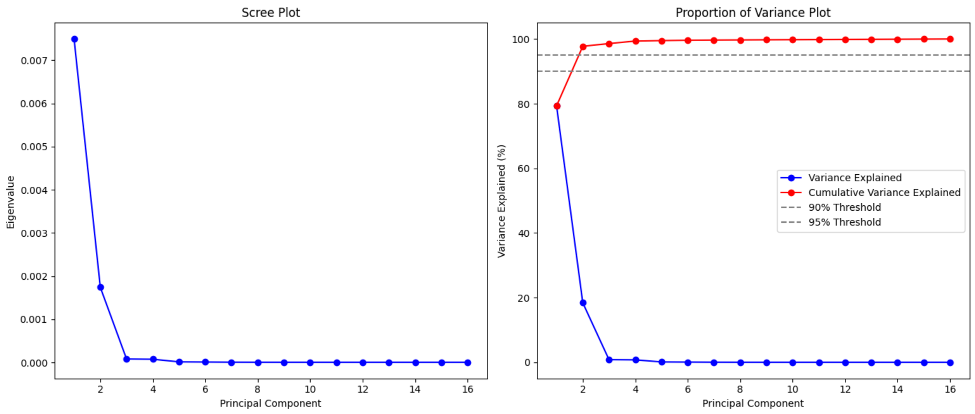

Admixed American/Latino Ancestry PC features plot

Figure S2: Principal component analysis (PCA) was performed to characterize genetic population structure across the cohort. The scatter plots display PCs 1–16, which capture the major axes of ancestry variation and show clear clustering of AFR, EUR, and AMR individuals. The accompanying scree plot illustrates the proportion of variance explained by each component, with a marked decline after PC11, indicating diminishing contribution of additional PCs to meaningful population structure. Based on these patterns, the first 11 PCs were selected as covariates for association analyses.
