# Supplementary material for: Multi-ancestry genome-wide association study in all of Us for primary open-angle glaucoma
Source: Sci Rep. 2026 Mar 17;16:13788. doi: 10.1038/s41598-026-43993-9 (PMC13129092; doi:10.1038/s41598-026-43993-9)
Supplement: Supplementary file 3 — Supplementary Material 3 [file 41598_2026_43993_MOESM3_ESM.pdf]

	Chromosome	Locus	Alleles 1	Alleles 2	SNP	Genes	Description of the gene	Category of the Gene	Beta	Standard_error	Odds Ratio	P_value	AF1 in Afr	AF2 in Afr	AF1 in Eur	AF2 in Eur	AF1 in Amr	AF2 in Amr	Functional consequen
African ancestry																			
	Chr5	176649091	A	G	rs73806060	TSPAN17	Tetraspanin 17	Protein coding	0.523	0.095582	5.4697	0.0000000451	0.9500	0.0501	NA	NA	NA	NA	intron_variant
	chr12	58659924	A	G	rs191915716	LOC10050	Uncharacterized LOC100506869, Long Intergenic Non-Protein Coding RNA 2388	RNA Gene (lncRNA)	0.802	0.13661	5.8729	0.0000000043	0.9800	0.0197	NA	NA	NA	NA	intron_variant
	chr12	58613818	C	G	rs186014623	LOC10050	RNA Gene (lncRNA)	RNA Gene (lncRNA)	0.771	0.13852	5.5678	0.0000000258	0.9800	0.0195	NA	NA	NA	NA	intron_variant
	Chr12	58667030	A	C	rs116501783	LOC10050	RNA Gene (lncRNA)	RNA Gene (lncRNA)	0.81	0.14461	5.5996	0.0000000215	0.9830	0.0174	NA	NA	NA	NA	intron_variant
	chr12	59677889	C	G	rs7301638	SLC16A7	Solute Carrier Family 16 Member 7	Protein coding	0.47	0.082899	5.6663	0.0000000146	0.9260	0.0736	NA	NA	0.9900	0.0102	transcript_variant
	chr13	104786704	GTATA	G	rs1488934513	LOC10798	Uncharacterized LOC107984606	RNA Gene (lncRNA)	1.06	0.18756	5.669	0.0000000144	0.9820	0.0181	NA	NA	NA	NA	intron_variant
	chr15	62333348	AGGGAG	A	rs2030912553	None			-17.2	3.0677	-5.5924	0.0000000224	0.9820	0.0181	NA	NA	NA	NA	Exon_variant
European																			
	chr1	165735511	AT	A	rs1651337149	TMCO1	Transmembrane And Coiled-Coil Domains 1	Protein coding	-0.364	0.051345	-7.0957	0.0000000000	0.1380	0.8620	0.1220	0.8780	0.1390	0.8610	intron_variant
	chr1	165717836	A	AT	rs1289423545	None			-0.35	0.052381	-6.69	0.0000000000	0.2160	0.7840	0.1170	0.8830	0.1500	0.8500	intron_variant
	chr1	165717968	G	A	rs4656461	None			-0.348	0.052426	-6.6358	0.0000000000	0.2160	0.7840	0.1170	0.8830	0.1510	0.8490	intron_variant
	chr1	165741852	T	G	rs4537525	TMCO1	Transmembrane And Coiled-Coil Domains 1	Protein coding	-0.344	0.052231	-6.582	0.0000000000	0.0750	0.9250	0.1170	0.8830	0.1300	0.8700	intron_variant
	chr1	165717915	A	G	rs4657474	None			-0.346	0.052471	-6.5915	0.0000000000	0.2160	0.7840	0.1170	0.8830	0.1510	0.8490	intron_variant
	chr1	165730355	CA	C	rs200155552	TMCO1	Transmembrane And Coiled-Coil Domains 1	Protein coding	-0.307	0.051827	-5.9232	0.0000000032	0.1460	0.8540	0.1250	0.8750	0.1420	0.8580	intron_variant
	chr1	165717722	G	A	rs4657472	None			-0.333	0.05554	-5.9868	0.0000000021	0.2140	0.7860	0.1030	0.8970	0.1460	0.8540	intron_variant
	chr1	165730173	C	A	rs71519271	TMCO1	Transmembrane And Coiled-Coil Domains 1	Protein coding	-0.323	0.053538	-6.0374	0.0000000016	0.0780	0.9220	0.1130	0.8870	0.1280	0.8720	intron_variant
	chr1	165730361	A	C	rs35862498	TMCO1	Transmembrane And Coiled-Coil Domains 1	Protein coding	-0.311	0.050892	-6.1195	0.0000000009	0.2150	0.7850	0.1280	0.8720	0.1940	0.8060	intron_variant
	chr1	165701721	T	C	rs1547725	LOC44070	Carbonic Anhydrase 14 Pseudogene	Pseudogene	-0.34	0.053383	-6.3754	0.0000000002	0.0446	0.9550	0.1110	0.8890	0.1260	0.8740	transcript_variant
	chr1	165736551	C	G	rs4603103	TMCO1	Transmembrane And Coiled-Coil Domains 1	Protein coding	-0.336	0.052359	-6.4261	0.0000000001	0.0790	0.9210	0.1170	0.8830	0.1310	0.8690	intron_variant
	chr1	165744003	A	G	rs10800153	TMCO1	Transmembrane And Coiled-Coil Domains 1	Protein coding	-0.337	0.052369	-6.4316	0.0000000001	0.0787	0.9210	0.1170	0.8830	0.1310	0.8690	intron_variant
	chr1	165725660	T	C	rs7524755	TMCO1	Transmembrane And Coiled-Coil Domains 1	Protein coding	-0.337	0.052255	-6.4517	0.0000000001	0.1330	0.8670	0.1180	0.8820	0.1350	0.8650	intron_variant
	chr1	165743723	G	A	rs4233408	TMCO1	Transmembrane And Coiled-Coil Domains 1	Protein coding	-0.337	0.05218	-6.4593	0.0000000001	0.2880	0.7120	0.1180	0.8820	0.1560	0.8440	intron_variant
	chr1	165722531	A	G	rs28504591	None			-0.341	0.052744	-6.4719	0.0000000001	0.0786	0.9210	0.1160	0.8840	0.1290	0.8710	intron_variant
	chr1	165750114	A	ATCTT	rs199626054	TMCO1	Transmembrane And Coiled-Coil Domains 1	Protein coding	-0.339	0.05227	-6.4827	0.0000000001	0.1140	0.8860	0.1180	0.8820	0.1360	0.8640	intron_variant
	chr1	165718428	A	C	rs12691499	None			-0.342	0.052579	-6.5069	0.0000000001	0.2290	0.7710	0.1170	0.8830	0.1510	0.8490	intron_variant
	chr1	165730167	C	A	rs546126577	TMCO1	Transmembrane And Coiled-Coil Domains 1	Protein coding	-0.34	0.052211	-6.5107	0.0000000001	0.1160	0.8840	0.1180	0.8820	0.1360	0.8640	intron_variant
	chr1	165730169	C	A	rs61800426	TMCO1	Transmembrane And Coiled-Coil Domains 1	Protein coding	-0.342	0.052267	-6.5397	0.0000000001	0.0789	0.9210	0.1170	0.8830	0.1510	0.8490	intron_variant
	chr1	165730171	C	A	rs113635272	TMCO1	Transmembrane And Coiled-Coil Domains 1	Protein coding	-0.342	0.052276	-6.5492	0.0000000001	0.0789	0.9210	0.1170	0.8830	0.1360	0.8640	intron_variant
	chr1	165745445	C	T	rs12133745	TMCO1	Transmembrane And Coiled-Coil Domains 1	Protein coding	-0.343	0.05229	-6.5544	0.0000000001	0.0729	0.9270	0.1170	0.8830	1.30 e-01	0.8700	intron_variant
	chr1	52498906	A	T	rs1320064399	TUT4	Terminal Uridylyl Transferase 4	Protein Coding	-33.1	5.8692	-5.6328	0.0000000177	0.9080	0.0921	0.9820	0.8800	1.30 e-01	0.8700	intron_variant
	chr1	165715470	T	C	rs6426936	ALDH9A1 (	Aldehyde Dehydrogenase 9 Family Member A1	Protein Coding	-0.343	0.053151	-6.457	0.0000000001	0.0323	0.9680	0.1120	0.8880	0.1290	0.8710	intron_variant
	chr1	165749742	C	A	rs7555523	TMCO1	Transmembrane And Coiled-Coil Domains 1	Protein coding	-0.339	0.052271	-6.4813	0.0000000001	0.1200	0.8800	0.1180	0.8820	0.1360	0.8640	intron_variant
	chr1	165774286	A	G	rs2790049	TMCO1-AS	TMCO1 Antisense RNA 1	RNA Gene (lncRNA)	-0.34	0.052214	-6.5035	0.0000000001	0.0859	0.9140	0.1180	0.8820	0.1300	0.8700	transcript_variant

	Chromosome	Locus	Alleles 1	Alleles 2	SNP	Genes	Description of the gene	Category of the Gene	Beta	Standard_error	Odds Ratio	P_value	AF1 in Afr	AF2 in Afr	AF1 in Eur	AF2 in Eur	AF1 in Amr	AF2 in Amr	Functional consequen
	chr1	165740093	T	C	rs7552679	TMCO1	Transmembrane And Coiled-Coil Domains 1	Protein coding	-0.341	0.052268	-6.5202	0.0000000001	0.0787	0.9210	0.1170	0.8830	0.1310	0.8690	intron_variant
	chr1	165703130	A	C	rs4656460	LOC44070	Carbonic Anhydrase 14 Pseudogene	Pseudogene	-0.336	0.053323	-6.3074	0.0000000003	0.0445	0.9550	0.1120	0.8880	0.1260	0.8740	intron_variant
	chr1	165718524	A	G	rs12691500	None			-0.343	0.052603	-6.5271	0.0000000001	0.2290	0.7710	0.1170	0.8830	0.1510	0.8490	intron_variant
	chr1	165726618	C	T	rs6660601	TMCO1	Transmembrane And Coiled-Coil Domains 1	Protein coding	-0.339	0.05226	-6.4905	0.0000000001	0.0739	0.9260	0.1170	0.8830	0.1300	0.8700	intron_variant
	chr1	165738686	T	C	rs6696454	TMCO1	Transmembrane And Coiled-Coil Domains 1	Protein coding	-0.341	5.23E-02	-6.529	0.0000000001	0.0738	0.9260	0.1170	0.8830	0.1300	0.8700	intron_variant
	chr1	165739396	A	AT	rs5778472	TMCO1	Transmembrane And Coiled-Coil Domains 1	Protein coding	-0.34	5.23E-02	-6.495	0.0000000001	0.0788	0.9210	0.1170	0.8830	0.1310	0.8690	intron_variant
	chr1	165740185	GT	G	rs201160181	TMCO1	Transmembrane And Coiled-Coil Domains 1	Protein coding	-0.33	5.25E-02	-6.2946	0.0000000003	0.1140	0.8860	0.1170	0.8830	0.1350	0.8650	intron_variant
	chr1	165745179	T	C	rs10918274	TMCO1	Transmembrane And Coiled-Coil Domains 1	Protein coding	-0.34	5.23E-02	-6.4956	0.0000000001	0.0729	0.9250	0.1170	0.8830	0.1300	0.8700	intron_variant
	chr1	165745505	C	G	rs10800154	TMCO1	Transmembrane And Coiled-Coil Domains 1	Protein coding	-0.337	5.24E-02	-6.4431	0.0000000001	0.0728	0.9270	0.1170	0.8830	0.1290	0.8710	intron_variant
	chr1	165746063	C	T	rs6426939	TMCO1	Transmembrane And Coiled-Coil Domains 1	Protein coding	-0.337	5.22E-02	-6.4604	0.0000000001	0.2880	0.7120	0.1180	0.8820	0.1560	0.8440	intron_variant
	chr1	165749294	A	AT	rs11409239	TMCO1	Transmembrane And Coiled-Coil Domains 1	Protein coding	-0.341	5.23E-02	-6.5182	0.0000000001	0.0755	0.9250	0.1170	0.8830	0.1300	0.8700	intron_variant
	chr1	165753409	T	G	rs6662839	TMCO1	Transmembrane And Coiled-Coil Domains 1	Protein coding	-0.342	5.23E-02	-6.536	0.0000000001	0.0749	0.9250	0.1170	0.8830	0.1300	0.8700	intron_variant
	chr1	165754533	A	G	rs10800155	TMCO1	Transmembrane And Coiled-Coil Domains 1	Protein coding	-0.337	5.22E-02	-6.4546	0.0000000001	0.1620	0.8380	0.1180	0.8820	0.1400	0.8600	intron_variant
	chr1	165763424	C	T	rs4657476	TMCO1	Transmembrane And Coiled-Coil Domains 1	Protein coding	-0.341	5.23E-02	-6.5236	0.0000000001	0.0738	0.9260	0.1170	0.8830	0.1300	0.8700	intron_variant
	chr1	165763863	A	G	rs7528177	TMCO1	Transmembrane And Coiled-Coil Domains 1	Protein coding	-0.341	5.23E-02	-6.5293	0.0000000001	0.0790	0.9210	0.1170	0.8830	0.1310	0.8690	intron_variant
	chr1	165765366	T	C	rs6668885	TMCO1	Transmembrane And Coiled-Coil Domains 1	Protein coding	-0.341	5.23E-02	-6.5195	0.0000000001	0.1140	0.8860	0.1170	0.8830	0.1360	0.8640	intron_variant
	chr1	165766938	C	T	rs4657477	TMCO1	Transmembrane And Coiled-Coil Domains 1	Protein coding	-0.343	5.23E-02	-6.5727	0.0000000000	0.0724	0.9280	0.1170	0.8830	0.1300	0.8700	intron_variant
	chr1	165767643	C	T	rs7518099	TMCO1	Transmembrane And Coiled-Coil Domains 1	Protein coding	-0.341	5.23E-02	-6.528	0.0000000001	0.0790	0.9210	0.1170	0.8830	0.1310	0.8690	intron_variant
	chr1	165769074	A	T	rs2251768	TMCO1	Transmembrane And Coiled-Coil Domains 1	Protein coding	-0.339	5.23E-02	-6.4885	0.0000000001	0.1140	0.8860	0.1180	0.8820	0.1360	0.8640	intron_variant
	chr1	165769226	G	C	rs2790052	TMCO1	Transmembrane And Coiled-Coil Domains 1	Protein coding	-0.341	5.23E-02	-6.5234	0.0000000001	0.0749	0.9250	0.1170	0.8830	0.1300	0.8700	intron_variant
	chr1	165770361	C	T	rs2814471	TMCO1	Transmembrane And Coiled-Coil Domains 1	Protein coding	-0.341	5.23E-02	-6.5324	0.0000000001	0.1140	0.8860	0.1170	0.8830	0.1360	0.8640	intron_variant
	chr3	134178625	G	A	rs886676876	RYK	Receptor Like Tyrosine Kinase	Protein Coding	-110	18.786	-5.8424	0.0000000052	NA	NA	NA	NA	NA	NA	intron_variant
	chr3	134177071	CAAACA	C	rs1184753876	RYK	Receptor Like Tyrosine Kinase	Protein Coding	-78.3	13.66	-5.7327	0.0000000099	NA	NA	NA	NA	NA	NA	intron_variant
	chr3	134178629	A	AT	rs1210832373	RYK	Receptor Like Tyrosine Kinase	Protein Coding	-37.5	6.5718	-5.7101	0.0000000113	NA	NA	0.9700	0.0297	NA	NA	
	chr6	132286913	T	C	rs144858466	None	None	None	0.415	0.073026	5.6893	0.0000000128	NA	NA	0.9940	0.0511	0.9750	0.0245	None
	chr6	132290313	A	G	rs17718841	None	None	None	0.392	0.069768	5.6231	0.0000000188	0.9850	0.0148	0.9430	0.0573	0.9140	0.0858	None
	chr9	33939861	AG	A	rs573630715	UBAP2	Ubiquitin Associated Protein 2	Protein Coding	-10.7	1.9637	-5.455	0.0000000490	0.9050	0.0952	0.8000	0.2000	NA	NA	intron_variant
Admixed Amrican/Latino																			
	chr8	138453425	C	CA	rs73717270	FAM135B	Family With Sequence Similarity 135 Member B	Protein Coding	1.25	0.22682	5.5253	0.0000000329	NA	NA	NA	NA	0.9660	0.0342	intron_variant
	chr11	65235773	C	T	rs138365802	SLC22A20	Solute Carrier Family 22 Member 20, Pseudogene	Pseudogene	0.914	0.15439	5.9188	0.0000000032	0.9890	0.0115	0.9370	0.0633	0.9660	0.0342	
	chr14	46485769	A	ATATAT	rs112720823	LINC00871	Long Intergenic Non-Protein Coding RNA 871	RNA gene	1.57	0.28838	5.4441	0.0000000521	0.9760	0.0237	0.9820	0.0184	0.9800	0.0198	intron_variant
	chr20	62509976	A	AT	rs1185300607	GATA5 <35	GATA Binding Protein 5	protein coding	-10	1.7772	-5.6381	0.0000000172	0.7830	0.2170	0.8440	0.1560	0.8300	0.1700	None
	chr22	19568156	A	C	rs111694222	LINC00895	Long Intergenic Non-Protein Coding RNA 895	RNA gene	1.26	0.21535	5.867	0.0000000044	0.9310	0.0692	0.9900	NA	NA		0.0102	transcript_variant

Table S2. Summary of ancestry-specific genome-wide significant and suggestive variants identified in the study.This table presents all loci identified across the African (AFR), European (EUR), and Admixed American (AMR) ancestry groups, including variant identifiers, chromosomal positions, effect alleles, nearest genes, functional annotations, and association statistics from the GWAS. Each entry includes the corresponding β coefficient, standard error, odds ratio, p-value, and allele frequencies where available. Gene annotations reflect the nearest or most biologically relevant candidate gene for each locus. Sheet titles indicate the ancestry group or dataset source.
