# Supplementary material for: Multi-ancestry genome-wide association study in all of Us for primary open-angle glaucoma
Source: Sci Rep. 2026 Mar 17;16:13788. doi: 10.1038/s41598-026-43993-9 (PMC13129092; doi:10.1038/s41598-026-43993-9)
Supplement: Supplementary file 4 — Supplementary Material 4 [file 41598_2026_43993_MOESM4_ESM.pdf]

Chromosome	Locus	Alleles 1	Alleles 2	SNP	Genes	Description of the gene	Category of the Gene	Beta	Standard_error	Odds Ratio	P_value	Coherance Q	I2 percent
chr21	44435042	G	A	rs41277556	TRPM2	Transient Receptor Potential Cation Channel Subfamily M Member 2	Protein Coding	0.17781	0.032536	5.465	0.0000000462	0.5236	0.0000
chr20	34860106	A	AG	rs896085411	GGT7	Gamma-Glutamyltransferase 7	Protein Coding	9.77	1.73	5.63	0.0000000170	0.7016	0.0000
Chr16	79722952			rs540492542	MAFTRR,LOC105371356	MAF Transcriptional Regulator RNA	RNA gene	1.45	0.264	5.485	0.0000000412	0.9801	0.0000
chr15	62333348	AGGGAG	A	rs2030912553	None			-17.2	3.0677	-5.5924	0.0000000224	0.0000	0.0000
chr13	104786704	GTATA	G	rs1488934513	LOC107984606	Uncharacterized LOC107984606	RNA Gene (lncRNA)	1.06	0.18756	5.669	0.0000000144	0.0000	0.0000
chr12	58659924	A	G	rs191915716	LOC100506869, LINC02388	Uncharacterized LOC100506869,Long Intergenic Non-Protein Coding RNA 2388	RNA Gene (lncRNA)	0.802	0.13661	5.8729	0.0000000043	0.0000	0.0000
chr12	58613818	C	G	rs186014623	LOC100506869, LINC02388	RNA Gene (lncRNA)	RNA Gene (lncRNA)	0.771	0.13852	5.5678	0.0000000258	0.0000	0.0000
Chr12	58667030	A	C	rs116501783	LOC100506869, LINC02388	RNA Gene (lncRNA)	RNA Gene (lncRNA)	0.81	0.14461	5.5996	0.0000000215	0.0000	0.0000
chr12	59677889	C	G	rs7301638	SLC16A7	Solute Carrier Family 16 Member 7	Protein coding	0.47	0.082899	5.6663	0.0000000146	0.0000	0.0000
chr12	59644358	G	A	rs58475293	SLC16A7	Solute Carrier Family 16 Member 7	Protein coding	0.446413	0.079412	5.621	0.0000000189	0.0000	0.0000
chr12	59643365	G	A	rs17122770	SLC16A7	Solute Carrier Family 16 Member 7	Protein coding	0.439241	0.079854	5.502771	0.0000000373	0.0082	0.0000
chr8	14551478	T	TAG	rs1172455560	SGCZ	Sarcoglycan Zeta	Protein coding	-2.64702	0.465863	-5.6989	0.0000000120	0.4573	0.0000
chr6	132286913	T	C	rs144858466	None	None	None	0.397	0.07	5.66	0.0000000143	0.7902	0.0000
chr5	25960795	T	TATATATAT	rs1491524026	None	None	None	0.730491	0.130059	5.616632	0.0000000195	5.4900	63.6100
Chr5	176649091	A	G	rs73806060	TSPAN17	Tetraspanin 17	Protein coding	0.523	0.095582	5.4697	0.0000000451	0.0000	0.0000
chr3	134178625	G	A	rs886676876	RYK	Receptor Like Tyrosine Kinase	Protein Coding	-110	18.786	-5.8424	0.0000000052	0.0000	0.0000
chr3	134177071	CAAACA	C	rs1184753876	RYK	Receptor Like Tyrosine Kinase	Protein Coding	-78.3	13.66	-5.7327	0.0000000099	0.0000	0.0000
chr3	134178629	A	AT	rs1210832373	RYK	Receptor Like Tyrosine Kinase	Protein Coding	-37.5	6.5718	-5.7101	0.0000000113	0.0000	0.0000
chr1	165701721	T	C	rs1547725	LOC440700	Carbonic Anhydrase 14 Pseudogene	Pseudogene	-0.26111	0.045213	-5.77517	0.0000000077	8.0136	75.0423
chr1	165703130	A	C	rs4656460	LOC440700	Carbonic Anhydrase 14 Pseudogene	Pseudogene	-0.26103	0.045141	-5.78257	0.0000000074	7.3883	72.9302
chr1	165715470	T	C	rs6426936	ALDH9A1 35kb up	Aldehyde Dehydrogenase 9 Family Member A1	Protein Coding	-0.27269	0.046013	-5.92627	0.0000000031	7.4654	73.2097
chr1	165717636	T	C	rs4657471	None			-0.25668	0.042517	-6.03714	0.0000000016	8.7870	77.2390
chr1	165722531	A	G	rs28504591	None			-0.24341	0.042845	-5.68108	0.0000000134	10.8534	81.5725
chr1	165725660	T	C	rs7524755	TMCO1	Transmembrane And Coiled-Coil Domains 1	Protein coding	-0.24996	0.039494	-6.32916	0.0000000002	7.7930	74.3360
chr1	165726618	C	T	rs6660601	TMCO1	Transmembrane And Coiled-Coil Domains 1	Protein coding	-0.25749	0.042538	-6.05323	0.0000000014	8.3884	76.1574
chr1	165730167	C	A	rs546126577	TMCO1	Transmembrane And Coiled-Coil Domains 1	Protein coding	-0.27762	0.046318	-5.99376	0.0000000021	7.4972	73.3233
chr1	165730167	A	C	rs546126577	TMCO1	Transmembrane And Coiled-Coil Domains 1	Protein coding	-0.25756	0.046694	-5.51593	0.0000000347	13.7361	85.4398
chr1	165730169	C	A	rs61800426	TMCO1	Transmembrane And Coiled-Coil Domains 1	Protein coding	-0.24612	0.042459	-5.79664	0.0000000068	10.1681	80.3306
chr1	165730171	CAAACA	C	rs1184753876	RYK	Receptor Like Tyrosine Kinase	Protein Coding	-0.24397	0.042512	-5.73887	0.0000000095	10.9781	81.7819
chr1	165730173	C	A	rs71519271	TMCO1	Transmembrane And Coiled-Coil Domains 1	Protein coding	-0.23865	0.043137	-5.53237	0.0000000316	7.4726	73.2356
chr1	165730355	CA	C	rs200155552	TMCO1	Transmembrane And Coiled-Coil Domains 1	Protein coding	-0.23081	0.038805	-5.94811	0.0000000027	6.9285	71.1339
chr1	165730361	A	C	rs35862498	TMCO1	Transmembrane And Coiled-Coil Domains 1	Protein coding	-0.22813	0.036143	-6.31182	0.0000000003	7.7999	74.3587
chr1	165735511	AT	A	rs1651337149	TMCO1	Transmembrane And Coiled-Coil Domains 1	Protein coding	-0.26087	0.039039	-6.68225	0.0000000000	11.4810	82.5799
chr1	165736551	AT	A	rs1651337149	TMCO1	Transmembrane And Coiled-Coil Domains 1	Protein coding	-0.23975	0.042535	-5.63662	0.0000000173	10.3301	80.6390
chr1	165738686	T	C	rs6696454	TMCO1	Transmembrane And Coiled-Coil Domains 1	Protein coding	-0.25705	0.042585	-6.03611	0.0000000016	8.8630	77.4342
chr1	165739396	A	AT	rs5778472	TMCO1	Transmembrane And Coiled-Coil Domains 1	Protein coding	-0.24295	0.042494	-5.71729	0.0000000108	10.3865	80.7442
chr1	165740093	T	C	rs7552679	TMCO1	Transmembrane And Coiled-Coil Domains 1	Protein coding	-0.24383	0.042485	-5.7391	0.0000000095	10.4744	80.9059
chr1	165741852	T	G	rs4537525	TMCO1	Transmembrane And Coiled-Coil Domains 1	Protein coding	-0.25528	0.042555	-5.99872	0.0000000020	9.5286	79.0105
chr1	165744003	A	G	rs10800153	TMCO1	Transmembrane And Coiled-Coil Domains 1	Protein coding	-0.24124	0.042539	-5.67101	0.0000000142	10.1309	80.2585
chr1	165745179	T	C	rs10918274	TMCO1	Transmembrane And Coiled-Coil Domains 1	Protein coding	-0.25269	0.042565	-5.93654	0.0000000029	9.2520	78.3829
chr1	165745445	C	T	rs12133745	TMCO1	Transmembrane And Coiled-Coil Domains 1	Protein coding	-0.25751	0.042656	-6.03686	0.0000000016	8.8907	77.5046
chr1	165745505	C	G	rs10800154	TMCO1	Transmembrane And Coiled-Coil Domains 1	Protein coding	-0.25396	0.042704	-5.94709	0.0000000027	8.6004	76.7452
chr1	165749294	A	AT	rs11409239	TMCO1	Transmembrane And Coiled-Coil Domains 1	Protein coding	-0.25148	0.042578	-5.90637	0.0000000035	9.3194	78.5394
chr1	165753409	T	G	rs6662839	TMCO1	Transmembrane And Coiled-Coil Domains 1	Protein coding	-0.25343	0.042572	-5.95306	0.0000000026	9.2066	78.2764
chr1	165754533	A	G	rs10800155	TMCO1	Transmembrane And Coiled-Coil Domains 1	Protein coding	-0.22277	0.038824	-5.73785	0.0000000096	11.2095	82.1580
chr1	165763424	C	T	rs4657476	TMCO1	Transmembrane And Coiled-Coil Domains 1	Protein coding	-0.25599	0.042575	-6.01256	0.0000000018	8.7383	77.1122
chr1	165763863	A	G	rs7528177	TMCO1	Transmembrane And Coiled-Coil Domains 1	Protein coding	-0.24402	0.042447	-5.74869	0.0000000090	10.3829	80.7375
chr1	165765366	T	C	rs6668885	TMCO1	Transmembrane And Coiled-Coil Domains 1	Protein coding	-0.22457	0.04085	-5.49751	0.0000000385	12.8471	84.4323
chr1	165766938	C	T	rs4657477	TMCO1	Transmembrane And Coiled-Coil Domains 1	Protein coding	-0.25884	0.042637	-6.07092	0.0000000013	8.6819	76.9636
chr1	165767643	C	T	rs7518099	TMCO1	Transmembrane And Coiled-Coil Domains 1	Protein coding	-0.24402	0.042447	-5.74883	0.0000000090	10.3663	80.7067
chr1	165768467	C	A	rs546126577	TMCO1	Transmembrane And Coiled-Coil Domains 1	Protein coding	-0.25353	0.042576	-5.95489	0.0000000026	9.1572	78.1592
chr1	165769074	A	T	rs2251768	TMCO1	Transmembrane And Coiled-Coil Domains 1	Protein coding	-0.22365	0.040854	-5.47427	0.0000000439	12.7014	84.2537
chr1	165769226	G	C	rs2790052	TMCO1	Transmembrane And Coiled-Coil Domains 1	Protein coding	-0.25318	0.042567	-5.94779	0.0000000027	9.1256	78.0837
chr1	165770361	C	T	rs2814471	TMCO1	Transmembrane And Coiled-Coil Domains 1	Protein coding	-0.22501	0.040851	-5.50807	0.0000000363	12.8996	84.4957
chr1	165774286	A	G	rs2790049	TMCO1-AS1	TMCO1 Antisense RNA 1	RNA Gene (lncRNA)	-0.24403	0.041953	-5.81684	0.0000000060	9.7947	79.5808

Table S3. Meta-analysis results for genome-wide significant and suggestive loci associated with POAG.
This table summarizes the genome-wide meta-analysis results across all cohorts, listing each lead variant along with its chromosomal position, alleles, rsID, nearest gene, functional annotation, and effect estimates. For each SNP, the table reports the meta-analytic beta coefficient, standard error, odds ratio, and p-value derived from the fixed-effect model. Measures of cross-study heterogeneity are provided through Cochran's Q statistic and the I^2 percentage, which quantify variability in effect sizes not attributable to sampling error. All SNPs meeting genome-wide significance ($p < 5\times10^{-8}$) or suggestive significance thresholds are included.
