# Supplementary material for: Multi-ancestry genome-wide association study in all of Us for primary open-angle glaucoma
Source: Sci Rep. 2026 Mar 17;16:13788. doi: 10.1038/s41598-026-43993-9 (PMC13129092; doi:10.1038/s41598-026-43993-9)
Supplement: Supplementary file 5 — Supplementary Material 5 [file 41598_2026_43993_MOESM5_ESM.pdf]

Lead SNP	Chr	Position	Nearest Gene	External Study	External SNP	Distance/LD	Direction consistant	External-Beta	External-P value	Evidance type
rs1651337149	1	165735511	TMCO1	UK Biobank	rs4656461	r2=1	Yes	1.51	6e-14	LD-based significant support
rs1289423545	1	165717836	NA	UK Biobank	rs4656461	r2=1	Yes	1.51	6e-14	LD-based significant support
rs4656461	1	165717968	NA	UK Biobank	rs4656461	Exact	Yes	1.51	6e-14	Exact significant replication
rs4537525	1	165741852	TMCO1	UK Biobank	rs7524755	r2=1	Yes	1.37	8e-41	LD-based significant support
rs4657474	1	165717915	NA	UK Biobank	rs7524755	r2=1	Yes	1.37	8e-41	LD-based significant support
rs200155552	1	165730355	TMCO1	UK Biobank	rs7524755	r2=1	Yes	1.37	8e-41	LD-based significant support
rs4657472	1	165717722	NA	UK Biobank	rs7524755	r2=1	Yes	1.37	8e-41	LD-based significant support
rs71519271	1	165730173	TMCO1	UK Biobank	rs7524755	r2=1	Yes	1.37	8e-41	LD-based significant support
rs35862498	1	165730361	TMCO1	UK Biobank	rs7524755	r2=1	Yes	1.37	8e-41	LD-based significant support
rs1547725	1	165701721	LOC440700	UK Biobank	rs7524755	1Mbp	Yes	1.37	8e-41	Significant locus-level support
rs4603103	1	165736551	TMCO1	UK Biobank	rs7524755	r2=1	Yes	1.37	8e-41	LD-based significant support
rs10800153	1	165744003	TMCO1	UK Biobank	rs7524755	r2=1	Yes	1.37	8e-41	LD-based significant support
rs7524755	1	165725660	TMCO1	UK Biobank	rs7524755	Exact	Yes	1.37	8e-41	Exact significant replication
rs4233408	1	165743723	TMCO1	UK Biobank	rs12133745	r2=1	Yes	0.13	1e-71	LD-based significant support
rs28504591	1	165722531	NA	UK Biobank	rs12133745	r2=1	Yes	0.13	1e-71	LD-based significant support
rs199626054	1	165750114	TMCO1	UK Biobank	rs12133745	r2=1	Yes	0.13	1e-71	LD-based significant support
rs12691499	1	165718428	NA	UK Biobank	rs12133745	r2=1	Yes	0.13	1e-71	LD-based significant support
rs546126577	1	165730167	TMCO1	UK Biobank	rs12133745	r2=1	Yes	0.13	1e-71	LD-based significant support
rs61800426	1	165730169	TMCO1	UK Biobank	rs12133745	r2=1	Yes	0.13	1e-71	LD-based significant support
rs113635272	1	165730171	TMCO1	UK Biobank	rs12133745	r2=1	Yes	0.13	1e-71	LD-based significant support
rs12133745	1	165745445	TMCO1	UK Biobank	rs12133745	Exact	Yes	0.13	1e-71	Exact significant replication
rs1320064399	1	52498906	TUT4	NA	NA	NA	NA	NA	NA	No external support available

Lead SNP	Chr	Position	Nearest Gene	External Study	External SNP	Distance/LD	Direction consistant	External-Beta	External-P value	Evidance type
rs6426936	1	165715470	ALDH9A1 35kb up	UK Biobank	rs7555523	1 Mbp	Yes	1.37	1e-12	Significant locus-level support
rs7555523	1	165749742	TMCO1	UK Biobank	rs7555523	Exact	Yes	1.37	1e-12	Exact significant replication
rs2790049	1	165774286	TMCO1-AS1	UK Biobank	rs2790049	Exact	Yes	1.35	1e-62	Exact significant replication
rs7552679	1	165740093	TMCO1	UK Biobank	rs2790049	r2=1	Yes	1.35	1e-62	LD-based significant support
rs4656460	1	165703130	LOC440700	UK Biobank	rs2790049	1Mbp	Yes	1.35	1e-62	Significant locus-level support
rs12691500	1	165718524	NA	UK Biobank	rs2790049	r2=1	Yes	1.35	1e-62	LD-based significant support
rs6660601	1	165726618	TMCO1	UK Biobank	rs2790049	r2=1	Yes	1.35	1e-62	LD-based significant support
rs6696454	1	165738686	TMCO1	UK Biobank	rs10918274	r2=1	Yes	1.08	4e-12	LD-based significant support
rs5778472	1	165739396	TMCO1	UK Biobank	rs10918274	r2=1	Yes	1.08	4e-12	LD-based significant support
rs201160181	1	165740185	TMCO1	UK Biobank	rs10918274	r2=1	Yes	1.08	4e-12	LD-based significant support
rs10918274	1	165745179	TMCO1	UK Biobank	rs10918274	Exact	Yes	1.08	4e-12	Exact significant replication
rs10800154	1	165745505	TMCO1	UK Biobank	rs4657476	r2=1	Yes	0.69	9e-18	LD-based significant support
rs6426939	1	165746063	TMCO1	UK Biobank	rs4657476	r2=1	Yes	0.69	9e-18	LD-based significant support
rs11409239	1	165749294	TMCO1	UK Biobank	rs4657476	r2=1	Yes	0.69	9e-18	LD-based significant support
rs6662839	1	165753409	TMCO1	UK Biobank	rs4657476	r2=1	Yes	0.69	9e-18	LD-based significant support
rs10800155	1	165754533	TMCO1	UK Biobank	rs4657476	r2=1	Yes	0.69	9e-18	LD-based significant support
rs4657476	1	165763424	TMCO1	UK Biobank	rs4657476	Exact	Yes	0.69	9e-18	Exact significant replication
rs7528177	1	165763863	TMCO1	UK Biobank	rs7528177	Exact	Yes	0.31	6e-141	Exact significant replication
rs6668885	1	165765366	TMCO1	UK Biobank	rs4657477	Exact	Yes	0.72	3e-09	LD-based significant support
rs4657477	1	165766938	TMCO1	UK Biobank	rs4657477	Exact	Yes	0.72	3e-09	Exact significant replication
rs7518099	1	165767643	TMCO1	UK Biobank	rs7518099	Exact	Yes	1.32	2e-52	Exact significant replication
rs2251768	1	165769074	TMCO1	UK Biobank	rs7518099	r2=1	Yes	1.32	2e-52	LD-based significant support

rs2790052	1	165769226	TMCO1	UK Biobank	rs2814471	r2=1	Yes	1.39	8e-44	LD-based significant support
rs2814471	1	165770361	TMCO1	UK Biobank	rs2814471	Exact	Yes	1.39	8e-44	Exact significant replication
rs886676876	3	134178625	RYK	NA	NA	NA	NA	NA	NA	No external support available
rs1184753876	3	134177071	RYK	NA	NA	NA	NA	NA	NA	No external support available
rs1210832373	3	134178629	RYK	NA	NA	NA	NA	NA	NA	No external support available
rs73806060	5	176649091	Tetraspanin17	POAAG G African GWAS	rs73806060	Exact	No	-0.0891	0.196863	Not replicated (direction inconsistent)
rs144858466	6	132286913	NA	NA	NA	NA	NA	NA	NA	No external support available
rs17718841	6	132290313	NA	NA	NA	NA	NA	NA	NA	No external support available
rs73717270	8	138453425	FAM135B	NA	NA	NA	NA	NA	NA	No external support available
rs573630715	9	33939861	UBAP2	NA	NA	NA	NA	NA	NA	No external support available
rs138365802	11	65235773	SLC22A20P	UK Biobank	rs12808303	r2=1	Yes	1.12	4e-10	LD-based significant support
rs191915716	12	58659924	LOC100506869, LINC02388	POAAG G African GWAS	rs191915716	Exact	Yes	0.065	0.55	Directionally consistent (not significant)
rs186014623	12	58613818	LOC100506869, LINC02388	POAAG G African GWAS	rs186014623	Exact	Yes	0.068	0.53	Directionally consistent (not significant)
rs116501783	12	58667030	LOC100506869, LINC02388	POAAG G African GWAS	rs116501783	Exact	Yes	0.091	0.43	Directionally consistent (not significant)
rs7301638	12	59677889	SLC16A7	POAAG G African GWAS	rs7301638	Exact	Yes	0.041	0.45	Directionally consistent (not significant)
rs1488934513	13	104786704	LOC107984606	AFR GWAS mega analysis	rs2025739	In 1Mbp	Yes	0.33	3.69e-06	Significant locus-level support
**Lead SNP**	**Chr**	**Position**	**Nearest Gene**	**External Study**	**External SNP**	**Distance/LD**	**Direction consistant**	**External-Beta**	**External-P value**	**Evidance type**
rs112720823	14	46485769	LINC00871	NA	NA	NA	NA	NA	NA	No external support available
rs2030912553	15	62333348	NA	NA	NA	NA	NA	NA	NA	No external support available
rs1185300607	20	62509976	GATA5 <35kb	23and me	rs12373885	1Mbp	Yes	0.05	6e-10	Significant locus-level support
rs111694222	22	19568156	LINC00895	UK Biobank	rs34175429	1Mbp	Yes	1.14	9e-09	Significant locus-level support

Table S1: External validation was performed using published POAG GWAS summary statistics and variants identified through the GWAS Catalog. Exact significant replication was defined as $p < 0.05$ with consistent direction of effect. LD-based support was defined as $r^2 \geq 0.6$ using a matched reference panel. Locus-level support was defined as proximity within ±1 Mb of a previously reported POAG-associated variant.
